# Craniofacial Fibrous Dysplasia to Affect or Not the Optic Nerve in Long-Term Follow-Up of Three Cases

**DOI:** 10.7759/cureus.91072

**Published:** 2025-08-26

**Authors:** Toshihiko Matsuo, Takehiro Tanaka, Kiyoshi Yamada, Mitsuhiro Okano

**Affiliations:** 1 Department of Ophthalmology, Graduate School of Interdisciplinary Science and Engineering in Health Systems, Okayama University, Okayama University Hospital, Okayama, JPN; 2 Department of Pathology, Graduate School of Medicine, Dentistry, and Pharmaceutical Sciences, Okayama University, Okayama, JPN; 3 Department of Plastic and Reconstructive Surgery, Graduate School of Medicine, Dentistry, and Pharmaceutical Sciences, Okayama University, Okayama, JPN; 4 Department of Otorhinolaryngology, School of Medicine, International University of Health and Welfare, Narita, JPN

**Keywords:** computed tomography (ct) scan, craniofacial bone, fibrous dysplasia, goldmann perimetry, magnetic resonance imaging, monostotic, optic nerve, pathology, visual acuity, visual field

## Abstract

Fibrous dysplasia of the bone is characterized by immature fibrous bones of trabeculae and fibrovascular proliferation in the medulla. In this study, we report three consecutive patients with craniofacial fibrous dysplasia with or without optic nerve involvement. In Case 1, a 43-year-old man with blurred vision in the right eye at the first visit was well until the age of 54 years, when he came back with symptoms suggestive of paranasal sinusitis. Computed tomography scans disclosed a mucocele in the right sphenoid sinus and thickened bilateral ethmoid, sphenoid, and frontal bones. He underwent an emergency nasal endoscopic surgery to make a drainage opening to the sphenoid and ethmoid sinuses on the right side with incomplete success. The pathology of the resected tissue confirmed fibrous dysplasia. With intravenous antibiotics, he recovered from blepharoptosis, complete ophthalmoplegia, and visual acuity decrease on the right side. He was well until the age of 71 years when he had a self-limiting episode of visual field cloudiness caused by the right sphenoid sinus mucocele. At the age of 75 years, he developed abrupt vision loss to no light perception in the right eye. He underwent an open skull surgery to extirpate the sphenoid mucocele on the right side and died of an unknown cause two years later. In Case 2, a 29-year-old man had a two-week-long headache, and computed tomography scans revealed fibrous dysplasia in the bilateral sphenoid bones. Nasal biopsy at the spheno-ethmoid recess proved a pathological diagnosis of fibrous dysplasia. Goldmann perimetry showed normal visual fields in both eyes. He was followed every year by magnetic resonance imaging to maintain normal visual fields until the latest visit at the age of 41 years. In Case 3, a 12-year-old girl was referred to an ophthalmologist to check her vision. She had been diagnosed with fibrous dysplasia of the left maxillary bone at the age of six years by a dentist. She had a gingival resection on the left maxilla at the age of 15 years and had a left maxillary bone resection at 18 years at another hospital. One month after the resection, Goldmann perimetry showed superior peripheral field depression in the left eye, in contrast with the normal visual field in the right eye. She maintained the visual acuity of 1.5 in both eyes until the last visit at the age of 21 years. In fibrous dysplasia as a rare disease, functional and cosmetic problems, including vision problems, should be considered in a case-based approach.

## Introduction

Fibrous dysplasia of the bone is characterized by immature fibrous bones of trabeculae and fibrovascular proliferation in the medulla [1]. The involvement of one bone is designated as monostotic, and the involvement of multiple bones is polyostotic. The combination of polyostotic fibrous dysplasia with skin pigmentations as café-au-lait spots, and endocrinopathy, as precocious puberty in girls, is called McCune-Albright syndrome. Predominant locations involved with monostotic fibrous dysplasia are long bones, especially the femoral cervix, in addition to the ribs, mandibles, and craniofacial bones. Long bones with fibrous dysplasia are prone to pathological fractures and deformity with pain. The disease is not inherited but has a point mutation as somatic mosaicism in the gene *GNAS*, which encodes the α-subunit of the stimulatory G-protein Gs (Gsα) as part of G protein-coupled receptors [2].

Craniofacial involvement with fibrous dysplasia [3-5] is often detected by chance in the routine X-ray examinations for dental procedures. The diagnosis is reached by computed tomography scans, which show ground-glass appearance with low bony density compared with the normal bone [6,7]. In magnetic resonance imaging, fibrous dysplasia shows low signals in T1-weighted images, while inhomogeneous signals in T2-weighted images [1,6,7]. In the field of ophthalmology, optic nerve compression by the surrounding bones with fibrous dysplasia is the major concern [8-12]. The sphenoid, ethmoid, and maxillary bones have paranasal sinuses, and sinusitis induced by a blockage to the sinus entrance would serve as a predisposing factor for optic nerve compression [8,9]. In this study, we report three consecutive patients with craniofacial fibrous dysplasia seen by a single ophthalmologist at a single institution. As a rare disease, vision problems, as well as other functional and cosmetic problems, should be considered in a case-based approach.

## Case presentation

Case 1

**Figure 1:**
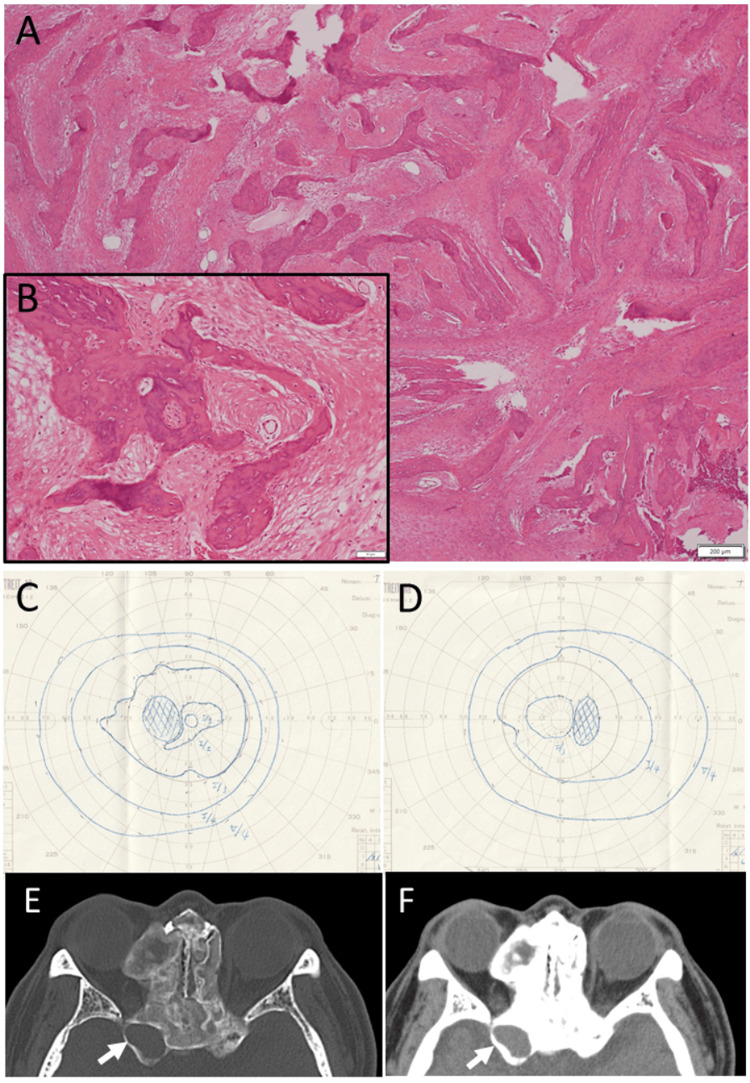
Case 1 - Pathology images, visual fields, and computed tomography scans at age 54-59 years Pathology of the resected tissue by emergency nasal endoscopic drainage at the right ethmoid and sphenoid sinuses at age 54 years, showing fibrous dysplasia (A: low magnification, B: high magnification). Note fibrous bone trabeculae and intramedullary fibrovascular tissue proliferation. Hematoxylin-eosin stain, white scale bar = 200 µm in A, and 50 µm in B. Goldmann perimetry at age 55, three months from the emergency surgery, revealing interior sensitivity reduction in the right eye with peripheral visual fields maintained in both eyes (C: left eye, D: right eye). Computed tomography scans at age 59 years, showing stable fibrous dysplasia in bilateral ethmoid and sphenoid bones with a protrusion into the right orbit. Note right sphenoid mucocele (arrows).

A 43-year-old man noticed blurred vision in the right eye and visited an ophthalmologist for the first time. The best-corrected visual acuity was 1.2 with highly myopic correction with spherical -11.0 diopters and cylindrical -1.5 diopters at the axis of 90 degrees in both eyes. Slit-lamp and fundus examinations revealed no cataract and normal optic discs in both eyes. He was healthy and had no past history. At the age of 54 years, he came back to an otolaryngologist because of nausea, vomiting, headache, right-sided eye pain, and diplopia. Computed tomography scans disclosed a mucocele in the right sphenoid sinus and an ethmoid protrusion in the right orbit in the background of thickened bilateral ethmoid, sphenoid, and frontal bones. White blood cell count increased to 14.6 x 10^3^/µL and serum C-reactive protein elevated to 11.5 mg/dL. He underwent an emergency nasal endoscopic surgery to make a drainage opening to the sphenoid and ethmoid sinuses on the right side, but the procedure ended up with incomplete success due to the bony thickening. The pathology of the resected tissue confirmed fibrous dysplasia (Figures 1A-1B). He received intravenous flomoxef 2 g daily and clindamycin 1,200 mg daily for five days and then had intravenous panipenem/betamipron (1:1) 2 g daily for five days in combination with intravenous hydrocortisone 500 mg daily for three days. He then had oral minocycline 200 mg daily for 7 days. Four days later from the emergency surgery, the best-corrected visual acuity was 0.09 in the right eye and 0.2 in the left eye. He had blepharoptosis and complete ophthalmoplegia on the right side. The pupil in the right eye showed an afferent pupillary defect, and the optic disc in the right eye showed temporal pallor. Three months later from the emergency surgery, the best-corrected visual acuity was 0.6 in the right eye and 1.0 in the left eye, and he showed normal eye movement and had no diplopia or blepharoptosis anymore. By Goldmann perimetry, he showed somewhat enlarged physiological scotomas in both eyes and maintained peripheral visual fields of both eyes (Figures 1C-1D). At the age of 59 years, computed tomography scans showed the same extent of fibrous dysplasia of bilateral ethmoid, sphenoid, and frontal bones (Figures 1E-1F). He had normal eye movement and had no diplopia. He underwent cataract surgeries with intraocular lens implantation in both eyes at the age of 61 years.

**Figure 2:**
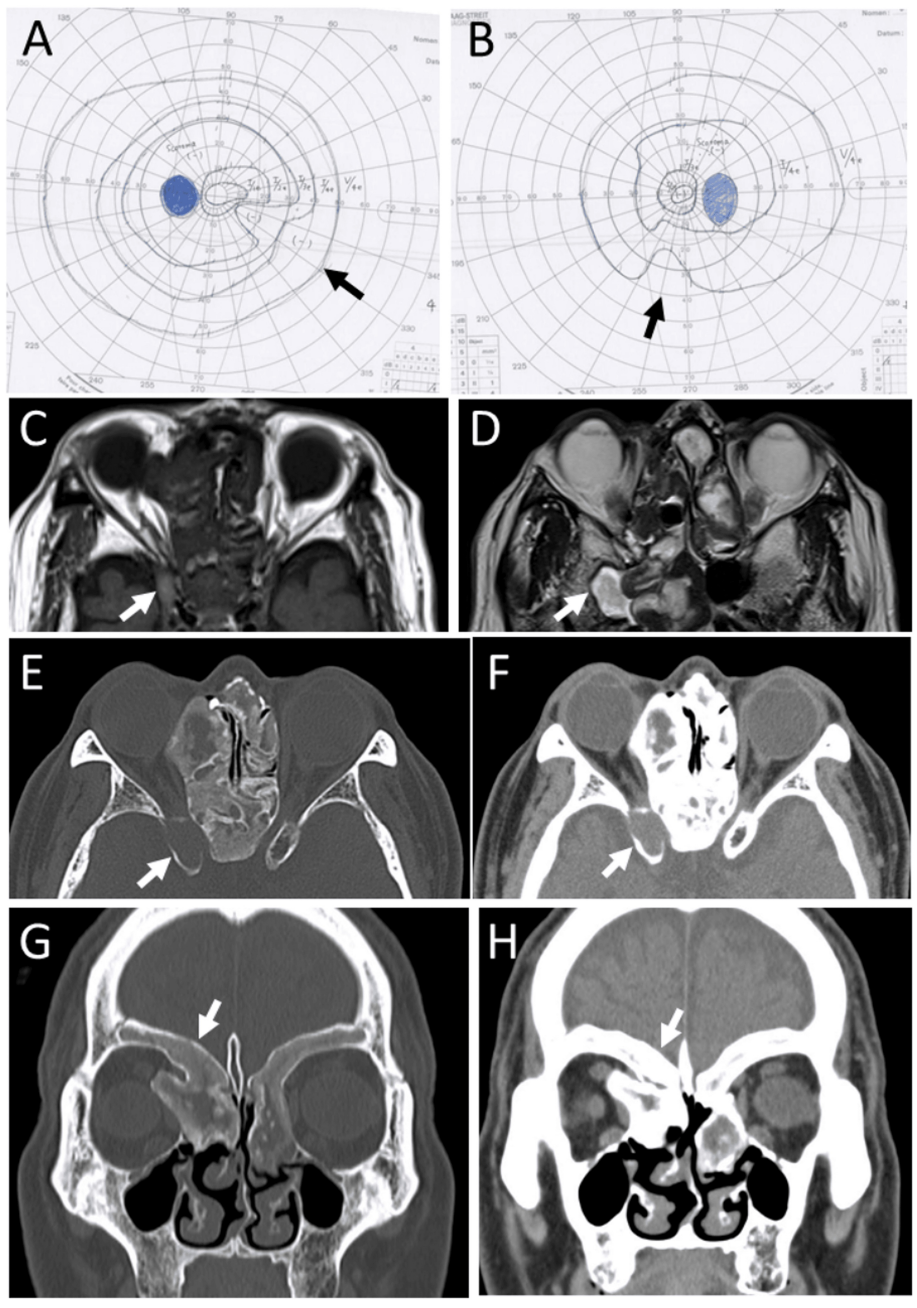
Case 1 - Visual fields, magnetic resonance imaging, and computed tomography scans at age 71 and 72 years Goldmann perimetry at age 71 years, showing inferior-nasal quadrantanopia-like changes (A: left eye, B: right eye). Magnetic resonance imaging (C: T1-weighted image, D: T2-weighted image), showing mucocele in the right sphenoid sinus (arrows). Computed tomography scans at age 72 years, four months from perimetric testing, showing right sphenoid sinus mucocele (arrows, axial images in E and F) and ethmoid bone protrusion (arrows, coronal images, G and H) in the right orbit. Note fibrous dysplasia also in bilateral frontal bones.

At the age of 71 years, he came back with a one-month-long symptom of lower-half visual field clouding in the right eye. The best-corrected visual acuity was 0.8 in the right eye and 1.2 in the left eye. Goldmann perimetry showed inferior-nasal quadrantanopia-like changes in both eyes (Figures 2A-2B). He had no diplopia or blepharoptosis. Magnetic resonance imaging (Figures 2C-2D) and computed tomography scans (Figures 2E-2H) showed a sphenoid sinus mucocele upper to the optic nerve on the right side, together with stable fibrous dysplasia of the ethmoid, sphenoid, and frontal bones on both sides. In consideration of the risk for an open skull surgery, he chose observation and was lost to follow-up at this hospital.

**Figure 3:**
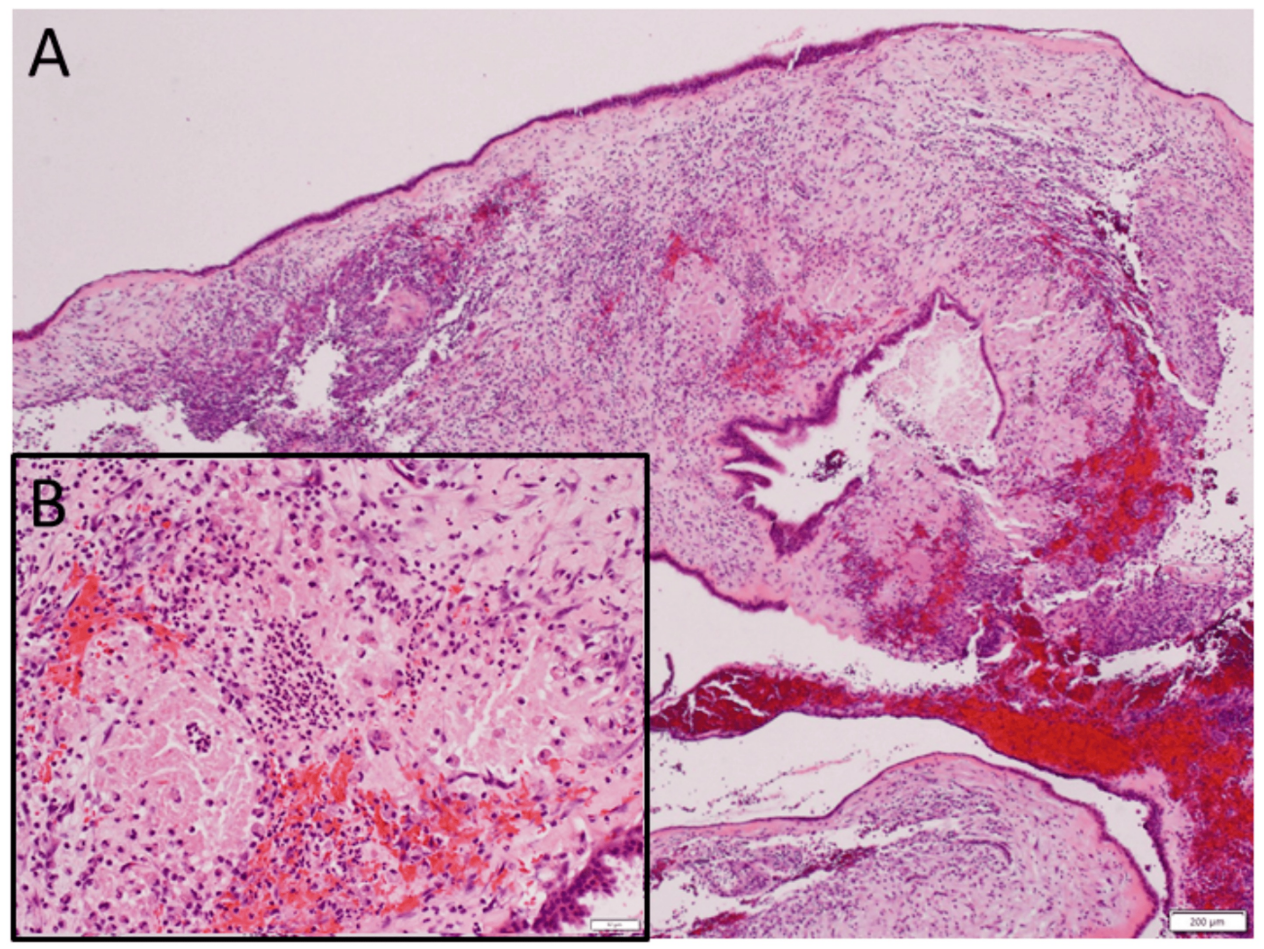
Case 1 - Pathology images at age 75 years Pathology of the resected tissue by open-skull right sphenoid sinus mucocele extirpation at age 75 years, showing infiltration with neutrophils in thickened mucosa (A: low magnification, B: high magnification). Hematoxylin-eosin stain, white scale bar = 200 µm in A and 50 µm in B.

At the age of 75 years, he developed abrupt vision loss to no light perception in the right eye and visited another hospital. He underwent an open skull surgery to extirpate the sphenoid mucocele on the right side and gained the visual acuity of light perception in the right eye. Pathological examinations showed the infiltration with neutrophils in the fibrovascular tissues of the sinus (Figures 3A-3B), indicative of a pyocele. He died of an unknown cause two years later.

Case 2

**Figure 4:**
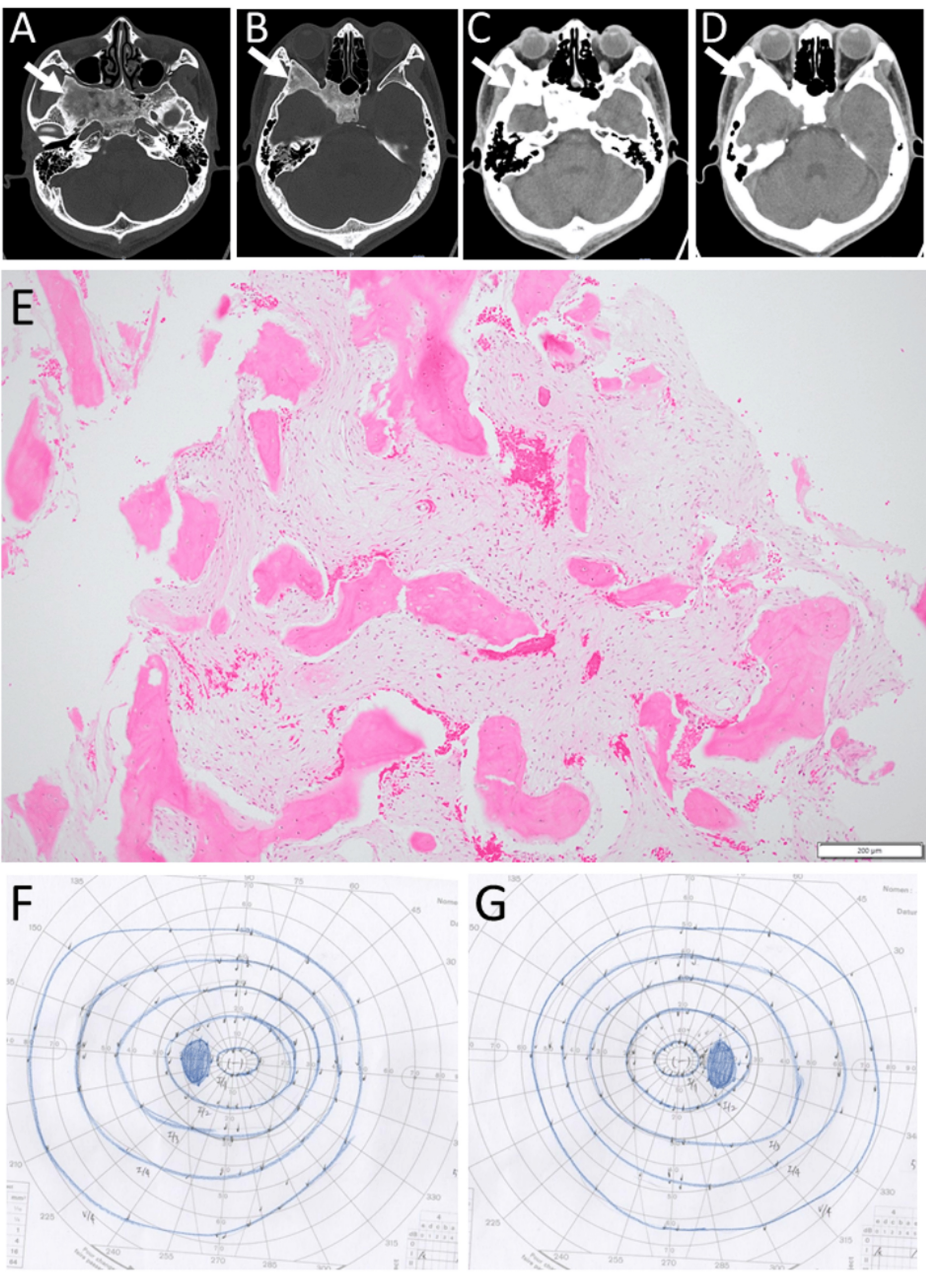
Case 2 - Computed tomography scans, pathology image, and visual fields at age 29 years Computed tomography scans (A-D) at age 29 years, showing fibrous dysplasia in bilateral sphenoid bones with more marked changes on the right side (arrows). Pathology of nasoscopic transmucosal biopsy (E) at the spheno-ethmoid recess on the right side, proving the diagnosis of fibrous dysplasia. Hematoxylin-eosin stain, white scale bar = 200 µm in E. Note fibrous bone trabeculae and intramedullary fibrovascular tissue proliferation. Goldmann perimetry, three months later, showing normal visual fields in both eyes (F: left eye, G: right eye).

**Figure 5:**
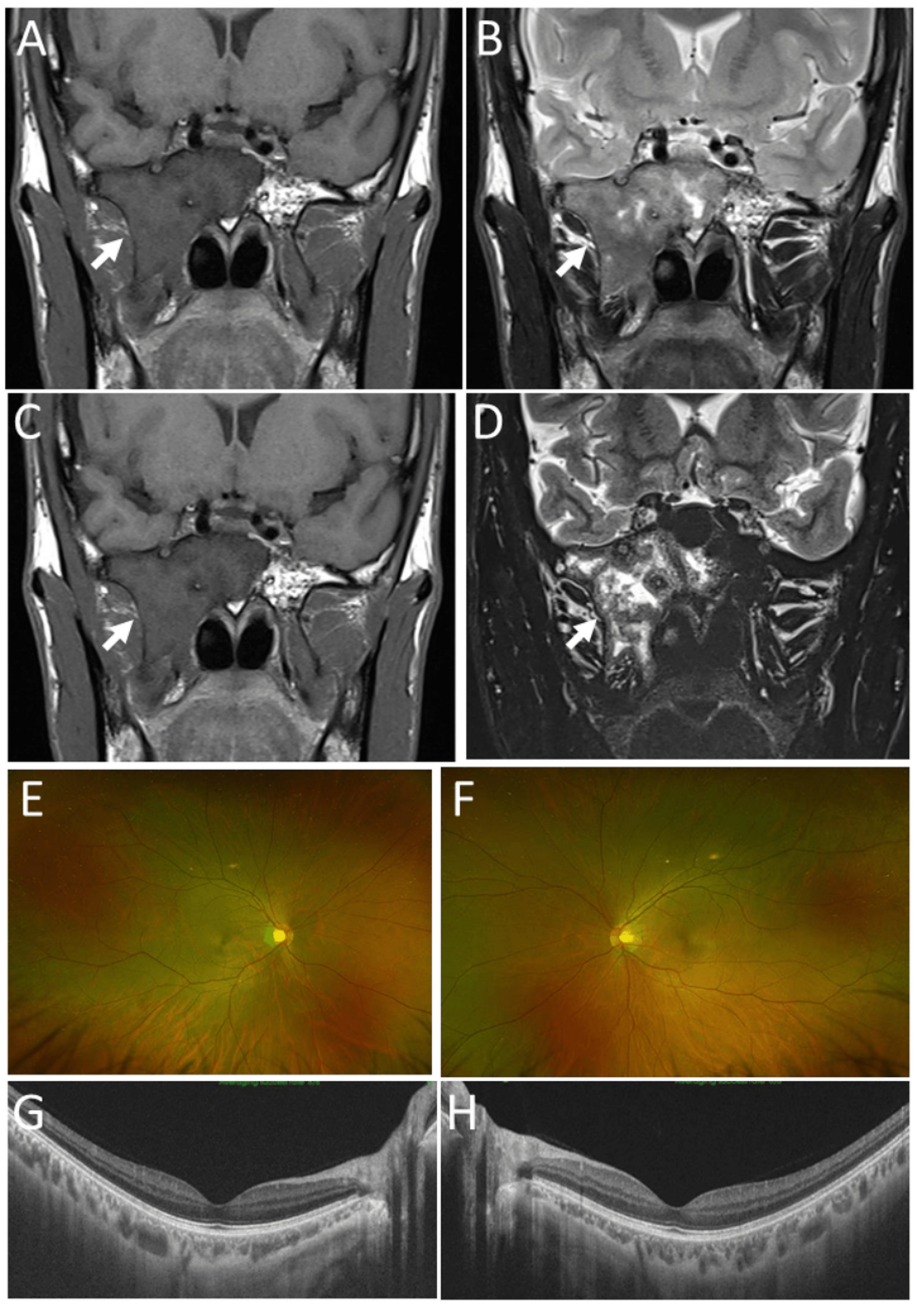
Case 2 - Magnetic resonance imaging at age 29 and 38 years and fundus photographs and optical coherence tomography at age 41 years Magnetic resonance images in coronal sections at annual follow-ups at age 29 years (A: T1-weighted image, B: T2-weighted image) and at age 38 years (C: T1-weighted image, D: T2-weighted image). Note no change of fibrous dysplasia, mainly on the right side (arrows). Wide-field fundus photographs (E: right eye, F: left eye), showing somewhat temporal pallor of the optic disc in the right eye (E), compared with the left eye (F) at age 41 years. Horizontal section images of optical coherence tomography are normal in both eyes (G: right eye, H: left eye).

A 29-year-old man had a two-week-long headache and visited a hospital. Computed tomography scans (Figures 4A-4D) revealed fibrous dysplasia in the bilateral sphenoid bones with a more marked lesion on the right side. A nasal biopsy at the spheno-ethmoid recess proved a pathological diagnosis of fibrous dysplasia (Figure 4E). He was otherwise healthy and had no past history. The best-corrected visual acuity was 1.5 in both eyes. Slit-lamp and fundus examinations revealed nothing abnormal. Goldmann perimetry showed the normal visual fields in both eyes (Figures 4F-4G). He was followed every year by magnetic resonance imaging (Figures 5A-5D) until the latest visit at the age of 41 years. He maintained visual acuity of 1.5 in both eyes with somewhat temporal pallor of the optic disc in the right eye (Figures 5E-5G), compared with the left eye (Figures 5F-5H).

Case 3

**Figure 6:**
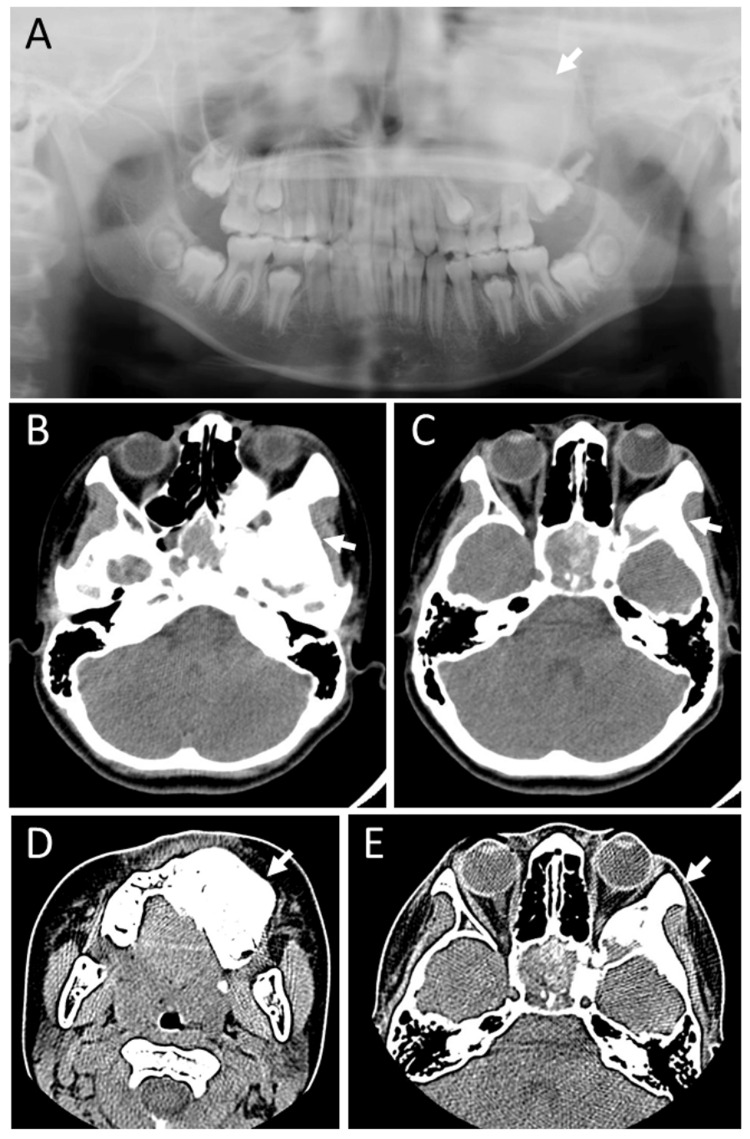
Case 3 - X-ray panoramic view and computed tomography scans at age 12 years Plain X-ray panoramic view at age 12 years, showing a high-density maxillary bone (arrow, A) on the left side, compared with the right side. Computed tomography scans (B-E), showing fibrous dysplasia in the right maxillary bone (arrows).

**Figure 7:**
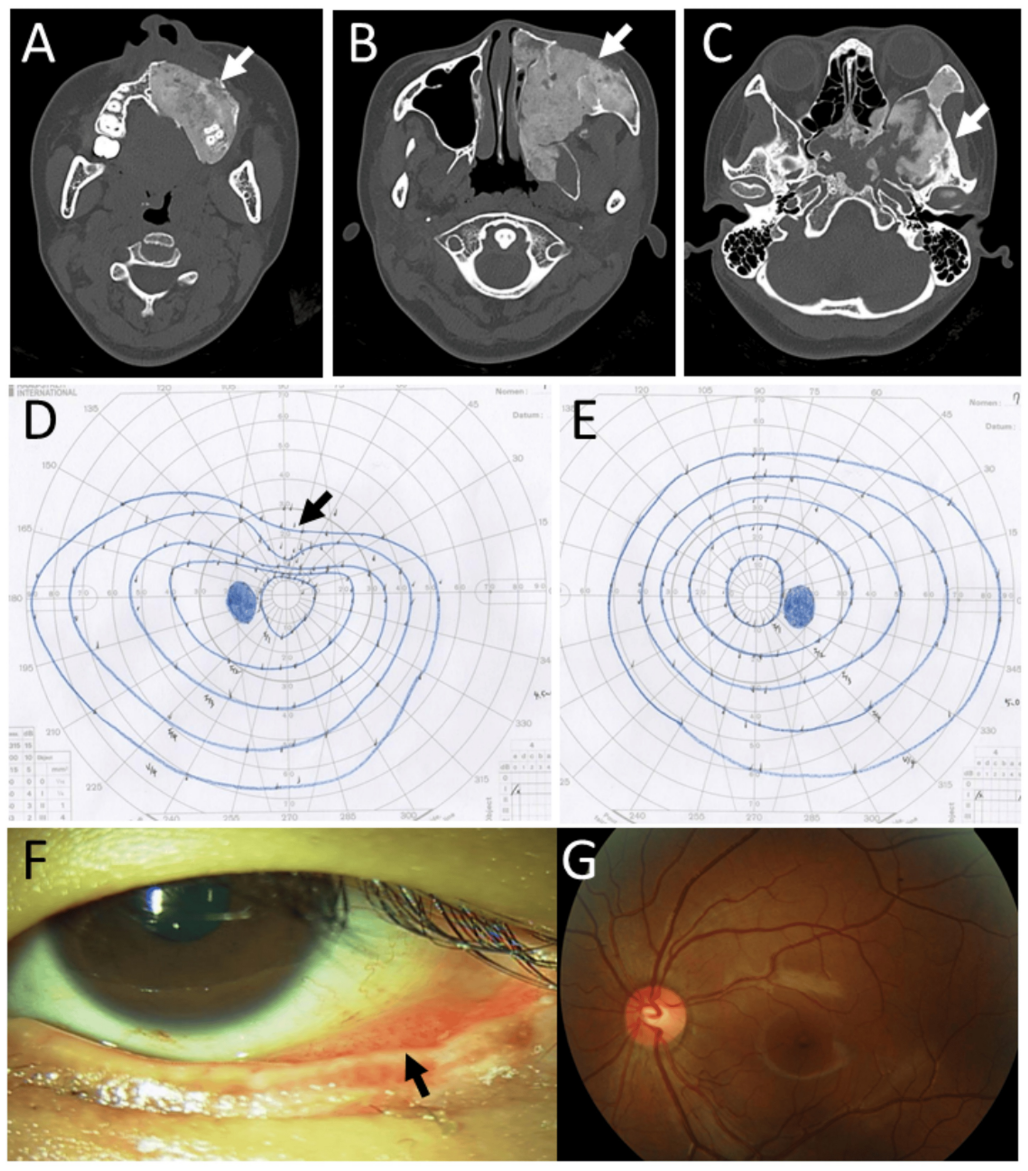
Case 3 - Computed tomography scans, visual fields, and slit-lamp and fundus photographs at age 18 years Computed tomography scans (A-C) at age 18 years, unchanged lesions of fibrous dysplasia in the left maxillary bone. Goldmann perimetry (D: left eye, E: right eye), showing superiorly depressed visual field (arrow) in the left eye (D), in contrast with normal visual field in the right eye (E). Bulbar conjunctiva in the left eye, showing lymphatic edema (arrow, F) and normal optic disc in the left eye (G).

**Figure 8:**
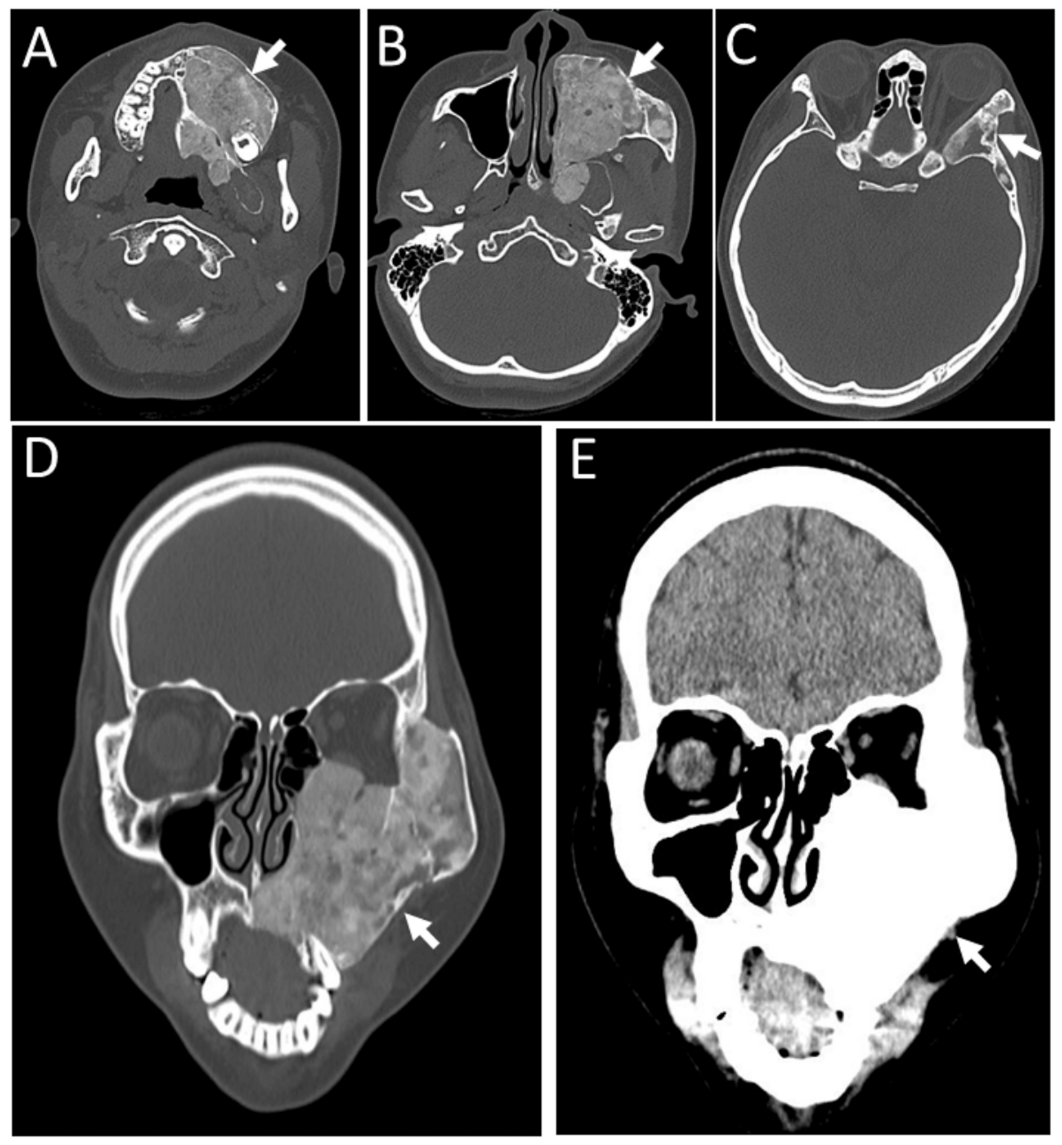
Case 3 - Computed tomography scans at age 21 years Computed tomography scans at age 21 years, showing unchanged lesions of fibrous dysplasia (arrows) in the left maxillary bone (A-C: axial images, D, E: coronal images).

A 12-year-old girl was referred to an ophthalmologist to check her vision. She had been diagnosed with fibrous dysplasia of the left maxillary bone since the age of six years by a dentist (Figures 6A-6E). Computed tomography scans showed the same lesion of fibrous dysplasia in the left maxillary bone (Figures 7A-7C). The best-corrected visual acuity was 1.5 in both eyes. Slit-lamp and fundus examinations were all normal in both eyes. She had a gingival resection on the left maxilla at the age of 15 years and had a left maxillary bone resection at the age of 18 years at another hospital. One month after the resection, Goldmann perimetry showed superior peripheral field depression in the left eye (Figure 7D), in contrast with the normal visual field in the right eye (Figure 7E). The eye showed lower eyelid ectropion on the left side with lymphatic edema (Figure 7F), which was attributed to the surgical intervention. The optic discs in both eyes were normal (Figure 7G). She maintained the visual acuity of 1.5 in both eyes until the last visit at the age of 21 years (Figures 8A-8E). Half a year later, she was reported to have resection of the left maxillary bone and insertion of an orbital floor plate in a different hospital.

## Discussion

Table 1 summarizes the three present patients with craniofacial fibrous dysplasia. Case 1 developed symptoms and signs of acute paranasal sinusitis at the age of 54 years and underwent an emergency nasal endoscopic surgery for drainage in the ethmoid and sphenoid sinuses on the right side. The diagnosis of fibrous dysplasia was made at this age. Although the surgical procedure was incomplete due to the thickened bones, he recovered almost completely from ophthalmoplegia, blepharoptosis, and visual acuity decrease on the right side, with the aid of antibiotic administrations. He had experienced an episode of blurred vision in the right eye 11 years previously, which might be a mild symptom of paranasal sinusitis. He had another episode with visual-field blurring at the age of 71 years, and the existing sphenoid sinus mucocele on the right side was considered responsible for the optic nerve compression. He chose observation at that time. Finally, at the age of 75 years, he had the third and last episode of the right sphenoid sinusitis, and this time, he underwent right sphenoid lesion extirpation by craniotomy. Case 2 was diagnosed as fibrous dysplasia in bilateral sphenoid bones on the occasion of headache at the age of 29 years and showed no symptoms or signs for 12 years. Case 3 was diagnosed as fibrous dysplasia of the left maxillary bone at the age of six years by a dentist. Superior visual field depression in the left eye and lymphatic conjunctival edema in the lower part of the left eye at the age of 18 years might be attributed to the surgical intervention for the left maxillary bone resection, which was done for a cosmetic purpose.

**Table 1:** Summary of three patients with craniofacial fibrous dysplasia LP, light perception

Case/Sex	Age at first ophthalmology visit	Symptoms or the reason for the visit	Age at diagnosis by imaging	Involved bones	Surgeries	Pathological diagnosis	Age at last visit	Eyes	Best-corrected visual acuity in decimals	Goldmann perimetry
1/Male	43 years	Blurred vision in the right eye	54 years with sinusitis	Bilateral sphenoid, ethmoid, frontal bones	Endoscopic drainage of ethmoid, sphenoid sinuses at 54 years, Right sphenoid mucocele extirpation at 75 years	Fibrous dysplasia at 54 years, Right sphenoid pyocele at 75 years	75 years	Right eye	1.2 at 43 years, 0.6 at 54 years, 0.8 at 71 years, LP at 75 years	Inferior-nasal quadrantanopia-like change at 71 years
								Left eye	1.5 at 43 years, 1.0 at 54 years, 1.2 at 71 years	Inferior-nasal quadrantanopia-like change at 71 years
2/Male	29 years	2-week-long headache	29 years	Bilateral sphenoid bones	Nasal biopsy at the spheno-ethmoid recess at 29 years	Fibrous dysplasia at 29 years	41 years	Right eye	1.5 at 29 and 41 years	Normal visual field at 29 and 41 years
								Left eye	1.5 at 29 and 41 years	Normal visual field at 29 and 41 years
3/Female	12 years	To check vision	6 years at the dentist visit	Left maxillary bone	Left maxillary gingival resection at 15 years, left maxillary bone resection at 18 years, left maxillary bone resection, and orbital floor plate insertion at 21 years	Not available	21 years	Right eye	1.5 at 12 and 21 years	Normal visual field at 18 years
								Left eye	1.5 at 12 and 21 years	Superior peripheral field depression at 18 years

Optic nerve compression is a well-known complication of paranasal sinusitis and is called sinusitis-induced optic neuropathy or rhinogenous optic neuropathy [13]. Usually, an emergency nasal endoscopic surgery is required to relieve the symptoms and to save the vision [14]. In case of craniofacial fibrous dysplasia, compression optic neuropathy could be caused by two situations: one is bone changes by fibrous dysplasia around the optic nerve canal, and the other is paranasal sinusitis caused by a narrowed opening of paranasal sinuses of the bones, which are involved with fibrous dysplasia [8-12]. According to the previous reports, it seems to be rare for craniofacial bones involved with fibrous dysplasia to exert a direct influence on the optic nerve canal [9,10]. In Case 1, optic nerve compression was caused repeatedly by the right sphenoid mucocele in the background of fibrous dysplasia of the bilateral sphenoid, ethmoid, and frontal bones. In the other two patients (Cases 2 and 3), no such paranasal sinusitis was found in the course of the follow-up. Based on the limited number of three patients in the present series of craniofacial fibrous dysplasia, paranasal sinusitis would be considered more responsible for optic nerve compression than optic nerve canal stenosis.

Nasal endoscopic surgical approaches to paranasal sinusitis in the background of fibrous dysplasia appear to be difficult [15,16], as shown in Case 1. In retrospect, the sphenoid sinus mucocele lesion in Case 1 would have required a surgical extirpation by craniotomy at the initial presentation at the age of 54 years but was indeed treated by the nasal endoscopic approach. However, it should also be emphasized that this patient maintained good vision and a healthy condition with no surgery for 21 years until the age of 75 years, when he finally underwent the surgical extirpation by a radical approach of craniotomy. In the previous report, a preventive measure or watchful waiting in the long-term yearly follow-up of patients with craniofacial fibrous dysplasia has been discussed to determine the timing for surgical intervention [17]. The present series of patients suggests that a case-based approach would be recommended in a rare disease like craniofacial fibrous dysplasia. The three case reports, in detail, would help clinicians make a decision to continue watchful waiting and ask an otolaryngologist to proceed to a nasal endoscopic surgery or craniotomy in craniofacial fibrous dysplasia.

## Conclusions

The long-term outcome and clinical features were described in detail in the three consecutive patients with craniofacial fibrous dysplasia: bilateral sphenoid, ethmoid, and frontal bones involved in Case 1, bilateral sphenoid bones in Case 2, and the left maxillary bone in Case 3. Pathological examinations of tissues obtained by the nasal endoscopic surgery and biopsy in two patients (Cases 1 and 2, respectively) showed immature fibrous bones of trabeculae and fibrovascular proliferation in the medulla, characteristic of fibrous dysplasia. Only one patient (Case 1) showed repeated episodes of compression optic neuropathy on the right side, which was attributable to the right sphenoid mucocele, while the other two patients developed no visual symptoms. Although there would be a limitation in a small series of three patients, the compression optic neuropathy might not be caused by fibrous dysplasia in itself in the bones that surround the optic nerve canal. Or rather, paranasal sinusitis, which is precipitated by craniofacial fibrous dysplasia, would be a key factor for developing the compression optic neuropathy, as shown in Case 1 of the present series. The location of craniofacial bone involvement with fibrous dysplasia is, of course, crucial to cause compression optic neuropathy, as the maxillary bone involvement in Case 3 would be less likely to influence the path of the optic nerve. The vision monitoring by ophthalmologists and craniofacial image monitoring by otolaryngologists are desirable in patients with craniofacial fibrous dysplasia.
